# Enhancing Anticancer Effect of Gefitinib across the Blood–Brain Barrier Model Using Liposomes Modified with One α-Helical Cell-Penetrating Peptide or Glutathione and Tween 80

**DOI:** 10.3390/ijms17121998

**Published:** 2016-11-29

**Authors:** Kuan-Hung Lin, Shu-Ting Hong, Hsiang-Tsui Wang, Yu-Li Lo, Anya Maan-Yuh Lin, James Chih-Hsin Yang

**Affiliations:** 1 Institute of Pharmacology, National Yang-Ming University, Taipei 112, Taiwan; kuanhunglin@outlook.com (K.-H.L.); cat820819@gmail.com (S.-T.H.); hsiangtsuiwang@gmail.com (H.-T.W.); 2 Faculty of Pharmacy, National Yang-Ming University, Taipei 112, Taiwan; 3 Department of Medical Research, Taipei Veterans General Hospital, Taipei 112, Taiwan; 4 Institute of Oncology, National Taiwan University, Taipei 106, Taiwan; chihyang@ntu.edu.tw

**Keywords:** gefitinib, blood–brain barrier (BBB), liposomes, peptides, lung cancer

## Abstract

Epidermal growth factor receptor (EGFR) tyrosine kinase inhibitors (TKI), such as gefitinib, have been demonstrated to effectively treat the patients of extracranial non-small cell lung cancer (NSCLC). However, these patients often develop brain metastasis (BM) during their disease course. The major obstacle to treat BM is the limited penetration of anticancer drugs across the blood–brain barrier (BBB). In the present study, we utilized gefitinib-loaded liposomes with different modifications to improve gefitinib delivery across the in vitro BBB model of bEnd.3 cells. Gefitinib was encapsulated in small unilamellar liposomes modified with glutathione (GSH) and Tween 80 (SUV-G+T; one ligand plus one surfactant) or RF (SUV-RF; one α-helical cell-penetrating peptide). GSH, Tween 80, and RF were tested by the sulforhodamine B (SRB) assay to find their non-cytotoxic concentrations on bEnd.3 cells. The enhancement on gefitinib across the BBB was evaluated by cytotoxicity assay on human lung adenocarcinoma PC9 cells under the bEnd.3 cells grown on the transwell inserts. Our findings showed that gefitinib incorporated in SUV-G+T or SUV-RF across the bEnd.3 cells significantly reduced the viability of PC9 cells more than that of free gefitinib. Furthermore, SUV-RF showed no cytotoxicity on bEnd.3 cells and did not affect the transendothelial electrical resistance (TEER) and transendothelial permeability of sodium fluorescein across the BBB model. Moreover, flow cytometry and confocal laser scanning microscopy were employed to evaluate the endocytosis pathways of SUV-RF. The results indicated that the uptake into bEnd.3 cells was mainly through adsorptive-mediated mechanism via electrostatic interaction and partially through clathrin-mediated endocytosis. In conclusion, cell penetrating peptide-conjugated SUV-RF shed light on improving drug transport across the BBB via modulating the transcytosis pathway(s).

## 1. Introduction

Non-small cell lung cancer (NSCLC) is one of the most common cancer types in the world. Current epidermal growth factor receptor (EGFR) tyrosine kinase inhibitors (TKIs) treatment, including gefitinib and afatinib, are highly effective to extracranial lung cancer patients with specific EGFR mutations [1]. Despite this, the major cause of death from lung cancer is due to metastases that are resistant to therapy. About 30% to 40% of patients with NSCLC have brain metastasis (BM) during the course of their disease [2]. The major hurdle to treat BM is the limited penetration of anticancer drugs across the blood–brain barrier (BBB). Clinical studies have reported that the cerebrospinal fluid-to-plasma ratio of gefitinib in patients with BM is only 0.3%–1.3% [3].

The BBB is a dynamic barrier protecting the brain against invading organisms and potential neurotoxins [4]. Specific tight junction features of the BBB allow low transport of antineoplastic agents to the brain [5]. Membrane pump transporters such as P-glycoprotein (P-gp), a protein associated with multidrug resistance (MDR), are one of the various mechanisms responsible for hampering drugs across the BBB. A new strategy to augment the permeation of anticancer drugs across the BBB for possible treatment of BM of NSCLC is thus in urgent need.

Gefitinib (trade name Iressa), an EGFR-TKI of first-generation, is the first line treatment for metastatic NSCLC with EGFR mutations (exon 19 deletion or exon 21 L858R mutation) [6]. Gefitinib reversibly binds to ATP binding site of tyrosine kinase and inhibits autophosphorylation of EGFR, thus blocking the downstream signaling of mitogen-activated protein kinase (MAPK) pathway and PI3K in cancer cells [7]. Gefitinib was initially effective for EGFR mutated brain metastases if the dose was increased to 1250 mg/day to provide a cerebrospinal fluid (CSF) concentration of in vitro IC_50_ [8]. However, clinical application for such high doses of gefitinib was hampered due to the side-effects in GI of diarrhea and skin rash [9]. Moreover, because of gefitinib′s low solubility in water and many solvents, the mixture of Cremophor EL, ethanol, and 5% dextrose is the common cosolvent for administration of gefitinib in liquid formulation. These vehicles can easily lead to a severe allergic reaction and possibly extract lots of plasticizer from polyvinyl chloride (PVC) plastic pipes and infusion bags [10]. Thus, the development of well-designed nanoparticles with multiple functions of modulating the blood–brain barrier, enhancing penetration into the cancer focus, and sustaining release of gefitinib at the tumor sites may provide a potential delivery platform for further improving BM therapy.

In the present study, liposomal delivery systems were attempted to enhance gefitinib penetration across the BBB. The in vitro BBB model was established by growing a monolayer of bEnd.3 cells on transwell inserts. The integrity of BBB was evaluated by measuring transendothelial electrical resistance (TEER) and transendothelial permeability.

Liposomes are composed of one or more lamellae of amphiphilic lipids, enclosing an internal aqueous compartment. Usually, the liposomal lipid bilayer consists of biocompatible and biodegradable lipids, present in biological membranes. One of the most recently applied strategies for drug delivery across the BBB endothelium using functionalized liposomes is based on the transcytosis mechanism using specific receptors on the luminal surface of cells [11]. Thus, our first approach is to use small unilamellar liposomes modified with glutathione (GSH) and Tween 80 (SUV-G+T; one ligand plus one surfactant).

Glutathione (GSH) receptors in the brain were largely localized in the white matter, more specifically in the neuroglial cells. GSH, an endogenous tripeptide, possesses a central nervous system (CNS) concentration up to 3 mM [12]. It has antioxidant-like characteristics and is readily transported across the BBB [13]. Recent reports have demonstrated that GSH can be used as a targeting ligand linked to PEGylated nanoparticles to improve delivery of therapeutics to CNS [14,15]. Among different approaches, the most advanced is 2B3-101, which is a liposomal doxorubicin formulation coated with GSH and PEG. This product has completed a Phase I/IIa clinical trial for multiple brain cancer indications, including glioma and brain metastases of breast cancer [14,15].

Polymeric nanoparticles modified with surfactants, such as polysorbates, have been demonstrated to help the particles permeate across the BBB [16] and improve brain targeting potential for nanoparticles [17]. Modification of nanoparticles with polysorbate 80 (Tween 80) has been reported to cross the BBB by enhancing the plasma adsorption of apolipoproteins, such as apolipoprotein E (ApoE) or B (ApoB), on the nanoparticle surface and thus enabling them to interact with the low density lipoproteins (LDL) receptor [18]. Such coating may enhance the nanoparticles internalization via receptor mediated endocytosis by brain endothelial cells [18,19]. In addition, since polysorbate 80 is also a P-gp modulator, it has been reported that nanoparticle coated with polysorbate 80 may escape the efflux by the P-gp transporter, and thus improving brain capillary endothelial cell uptake of nanoparticles [19,20]. In this study, liposomes are prepared with surface modification by GSH and/or Tween 80 to evaluate their BBB penetration and cytotoxicity on human lung adenocarcinoma PC-9 cells.

Furthermore, our second approach is to use liposomes conjugated with RF, one cell-penetrating peptide (CPP). Surface modulation of nanoparticles with CPPs assisted in endosomal escape and expedited their cellular internalization [21]. CPPs have multiple choice in sequence design [22]. A traditional CPP, the trans-activator of transcription (TAT) of HIV-1, has been extensively conjugated on the surface of drug-loaded nanoparticles to increase the transport efficiency across the BBB to the CNS [23]. However, drawbacks of TAT include non-human origin, immunogenicity, and low delivery efficiency [24]. Recent studies have explored a new CPP called RF from screening of the 16 amino acid peptide library [25,26,27,28]. The RF peptide has a α-helical structure with a high normal cell compatibility, similar cell penetrating and cancer cell death activities compared with those of TAT [25,27]. Additionally, RF showed a better cell selectivity than that of TAT. It showed lower uptake into 3T3-L1 cells, but higher uptake into human alveolar epithelial adenocarcinoma A549 cells and human cervical cancer HeLa cells [25]. This peptide was internalized via endocytosis, then surrounded inside endosomes or lysosomes, escaped from endosome, and consequently distributed around the nucleus [25]. Thus, RF possesses the potential to be conjugated into a delivery system with the advantages of high efficiency, low toxicity, and cell selectivity [25,28]. In this study, TAT was also evaluated for comparison. Based on the above understanding, we thus aim to utilize gefitinib-encapsulated PEGylated liposomes, which were surface modified with GSH and/or polysorbate 80 or conjugated to RF for enhancing gefitinib across the bEnd3 cells to exhibit their cytotoxicity on PC9.

## 2. Results

### 2.1. Determination of Encapsulation Efficiency (EE)%, Particle Size, and *ζ* Potential of PEGylated Liposomal Gefitinib

**Figure 1:**
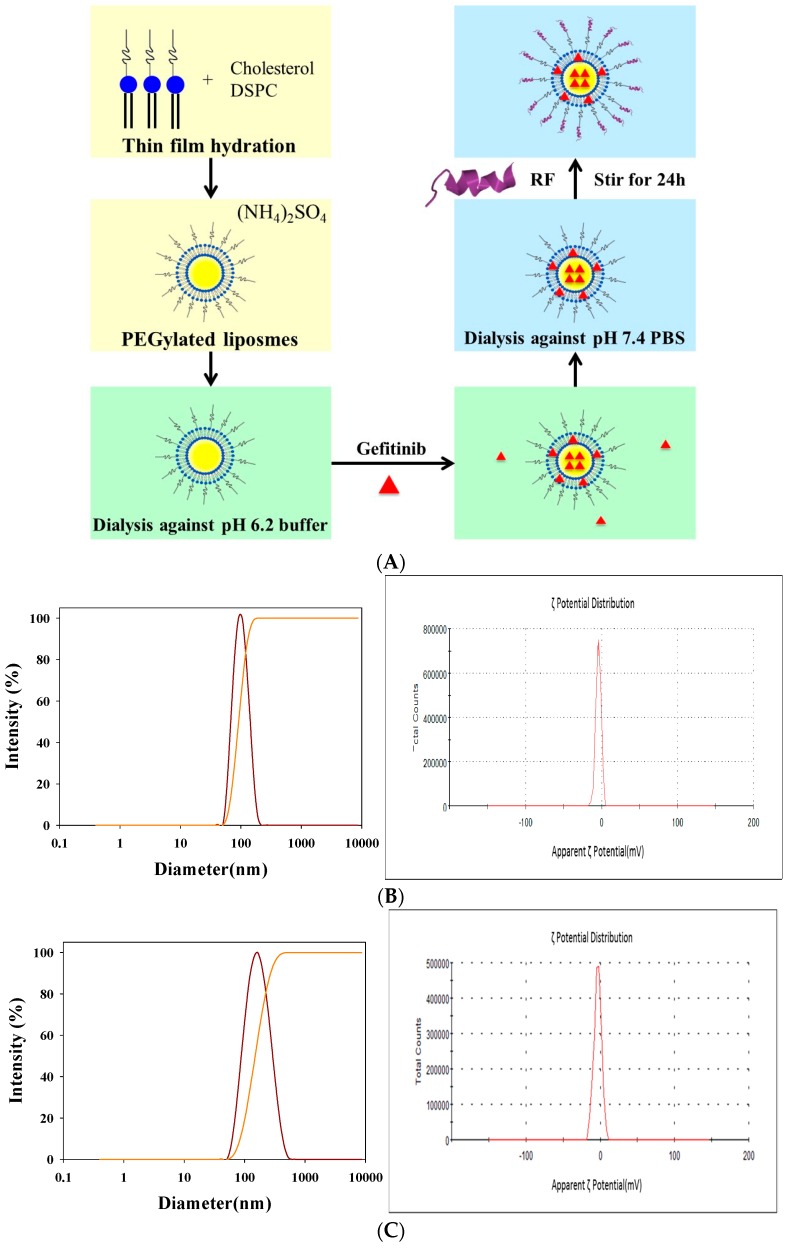

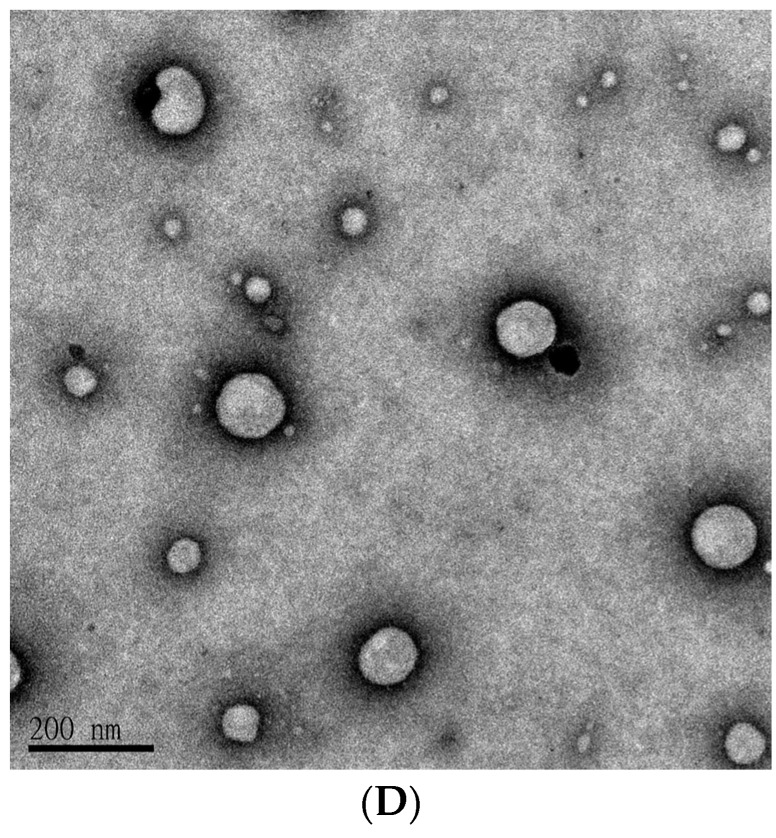
(**A**) A schematic diagram for the preparation of PEGylated liposomal delivery system of SUV-Mal and SUV-RF. Particle size distribution and ζ potential of PEGylated liposomes of: (**B**) SUV-G+T (SUV-GSH + Tween 80); and (**C**) SUV-RF. Transmission electron microscopic image of PEGylated liposomes of (**D**) SUV-RF. Bar = 200 nm.

Characteristics of PEGylated liposomes modified with GSH, Tween 80, GSH plus Tween 80, and RF were summarized in Table 1. A schematic graph displaying the formation of PEGylated liposomes conjugated with RF peptide is shown in Figure 1A. These liposomal preparations with or without modification were well-dispersed nanoparticles with sizes changed from 85.8 ± 3.7 nm for SUV-T (SUV-Tween 80) to 147.1 ± 3.9 nm for SUV-RF (Figure 1B,C; Table 1), with a polydispersity index about 0.1 (Table 1). The mean zeta potential of liposomes was ranged from −3.82 ± 0.85 to −1.70 ± 0.16 mV (*n* = 3; Table 1). The morphology of these liposomal dispersions was observed by transmission electron microscope (TEM). As demonstrated in Figure 1D, this population of liposomes SUV-RF displayed a diameter around 100 nm. These nanoparticles were close to spherical in shape (Figure 1D). Encapsulation efficiency (EE)% of these PEGylated liposomes was 86.70% ± 2.75%.

### 2.2. In Vitro Release of Gefitinib from Small Unilamellar Vesicle (SUV)

**Figure 2:**
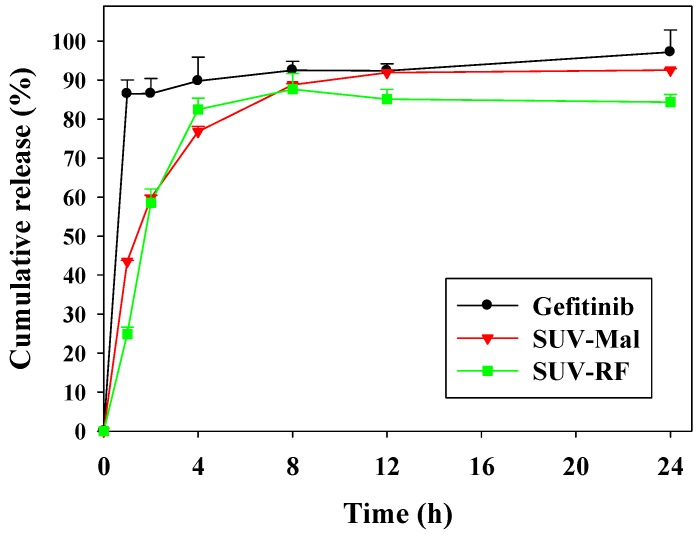
In vitro release of gefitinib from SUV-Mal and SUV-RF. The gefitinib release from liposomes or free drug was conducted in dialysis bag at pH 7.4 PBS with 2% Tween 80 at 37 °C.

The in vitro release experiments of gefitinib alone and gefitinib in SUV-Mal or conjugated with RF were investigated using a dynamic release assay under sink conditions, as shown in Figure 2. The assay was carried out via dialysis using a regenerated cellulose dialysis membrane with an initial gefitinib concentration of 20 µM, in 500 mL of phosphate-buffered saline (PBS) with 2% Tween 80 at 37 °C for 24 h. We found that a higher release percent from gefitinib alone was monitored in the initial phase compared with the gefitinib release from the SUV-Mal or SUV-RF (Figure 2). It was observed that 86.49% ± 3.54% of the amount of gefitinib was released after 1 h when compared with 24.86% ± 1.84% of gefitinib release for SUV-RF (Figure 2). After 24 h, the amount of gefitinib released from SUV-RF is 84.37% ± 1.97%, in contrast to 97.21% ± 5.65% of gefitinib being released in its free form (Figure 2).

### 2.3. The Blood–Brain Barrier (BBB) Barrier Integrity

**Figure 3:**
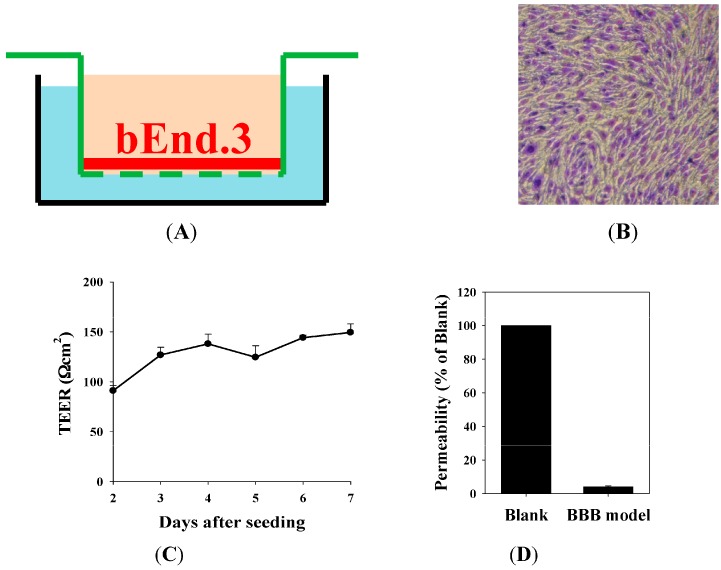
Establishment and barrier integrity of in vitro blood–brain barrier (BBB) model by growing bEnd.3 monolayer on transwell inserts. (**A**) Schematic of the in vitro BBB model; (**B**) Morphology of the BBB model stained with crystal violet. Representative images of intact cell monolayer were captured under a light microscope (magnification, 100×); (**C**) The TEER values were evaluated for seven days after seeding; (**D**) Permeability of fluorescein isothiocyanate (FITC)-dextran across the BBB model at Day 7. Values are the mean ± S.E.M. (*n* = 3).

The in vitro BBB model was constructed using bEnd.3 cells grown on Transwell inserts of 0.4 µm pore size (Figure 3A). Morphology of the BBB model was observed by staining bEnd.3 cells with crystal violet and cell image was captured under a Nikon inverted tissue culture microscope (Figure 3B). The bEnd.3 cells exhibited dense, uniform, and intact monolayer characteristics. The BBB barrier integrity was evaluated by TEER measurement and permeability study (Figure 3C,D). After seeding bEnd.3 cells on inserts for two days, TEER was 91.07 ± 5.08 Ω·cm^2^ (Figure 3C). After four-day culture, TEER reached 138.01 ± 9.70 Ω·cm^2^, which represented these monolayers were ready for the following experiments as a BBB model (Figure 3C). Consistently, the previous report also used the TEER value above 130 Ω·cm^2^ for their BBB model [29]. Furthermore, permeability percent of FITC-dextran (molecular weight 70,000) across the BBB was 4.02% ± 0.57% relative to that of FITC-dextran without the BBB (blank) (Figure 3D). According to the previous studies, these researchers also used FITC-dextran (MW 70,000) as a paracellular marker to evaluate the permeability of this marker across the BBB model [30,31,32]. The BBB barrier integrity was thus verified based on the morphology, TEER, and permeability studies.

### 2.4. Cytotoxicity of Tween 80, GSH, RF, TAT, and Gefitinib on bEnd.3 and/or PC9 Cells

**Figure 4:**
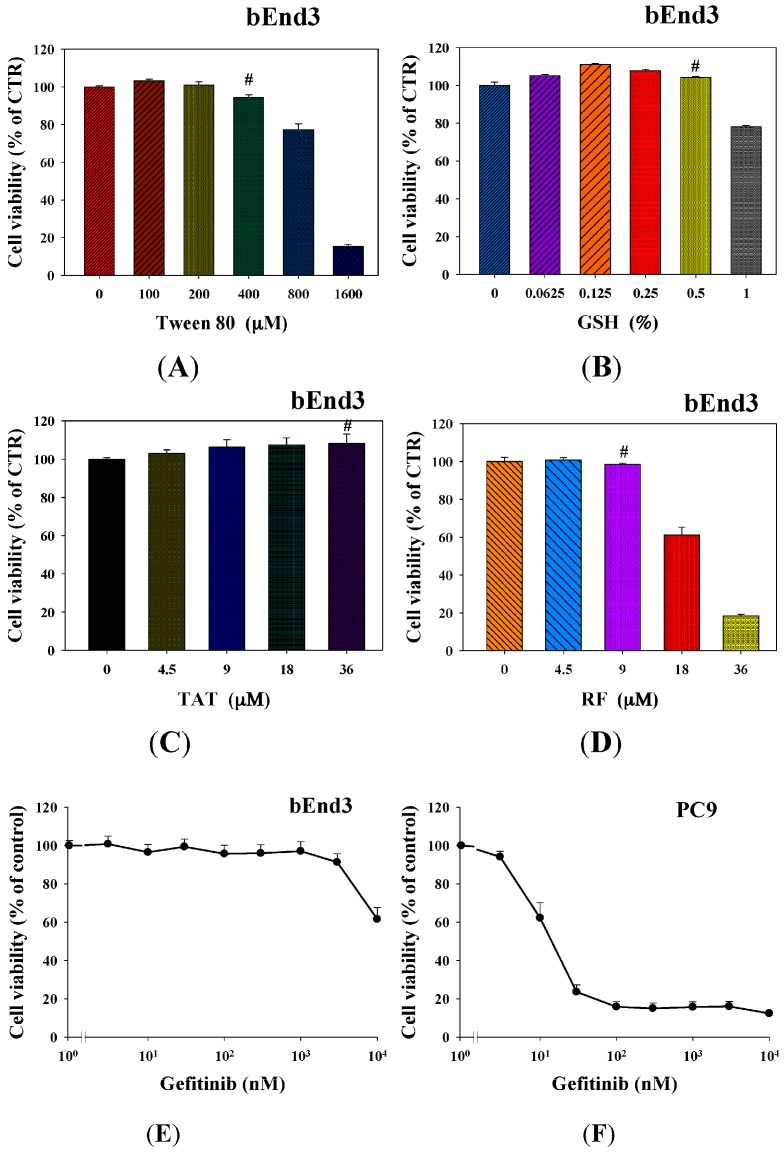
Cytotoxic effect of gefitinib, Glutathione (GSH), Tween 80, trans-acting activator of transcription (TAT), and RF on PC9 and bEnd.3 cells. bEnd.3 cells were cultured for 24 h with various concentrations of: (**A**) Tween 80; (**B**) GSH; (**C**) TAT; and (**D**) RF. Cell viability was determined using SRB assay. (**E**) bEnd.3 were cultured in various concentrations of gefitinib for 96 h. (**F**) PC-9 were cultured with various concentrations of gefitinib for 48 h. Cell viability was determined using the sulforhodamine B (SRB) assay. Values are the mean ± SEM. (*n* = 3). # represents the non-cytotoxic concentrations of GSH, Tween 80, TAT, and RF, which were used in the following experiments.

We tested the cytotoxicity of Tween 80, GSH, TAT, and RF on bEnd.3 cells by the sulforhodamine B (SRB) assay and found the concentrations of these compounds that maintained the viability of bEnd.3 cells over 90% (marked as #) were 400 µM, 0.5%, 36 and 9 µM, respectively (Figure 4A–D). Interestingly, more than 90% of bEnd.3 cells kept alive after treatment with gefitinib at 1 µM (Figure 4E). However, as we increased the concentrations of gefitinib to 10 µM, viability of bEnd.3 cells was significantly diminished to 60% (Figure 4E). Furthermore, we evaluated the cytotoxicity of gefitinib on PC9 cells and found that IC_50_ was 16.34 nM using a regression line for the plot with the linear scale in the *x*-axis (Figure 4F). Since our purpose was to verify if these liposomal formulations would enhance the cytotoxicity of gefitinib, we determined to use 15 nM of gefitinib for the following investigation. Notably, gefitinib at the concentration of 15 nM did not cause cytotoxicity to bEnd.3 cells (Figure 4E).

### 2.5. Cytotoxicity of Gefitinib in SUV-G, SUV-T, SUV-G+T across the BBB on PC9 Cells

**Figure 5:**
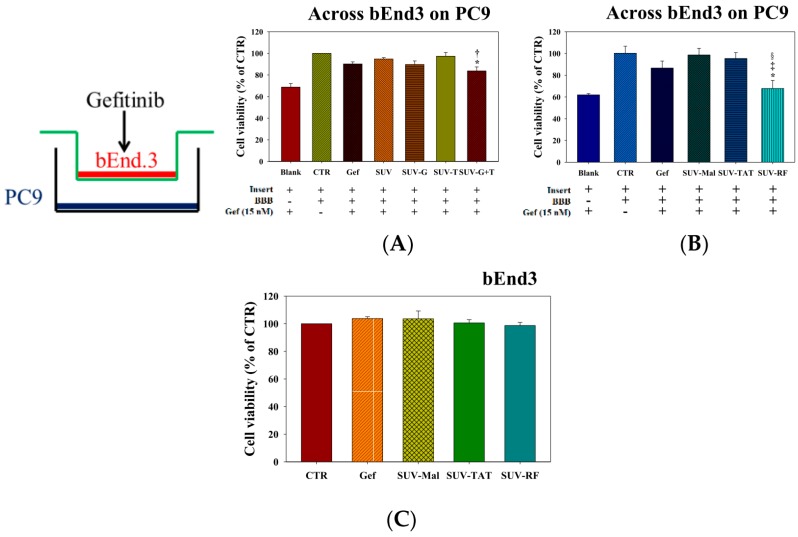
Cytotoxic effects of different liposomal gefitinib formulations across the BBB on PC9: (**A**) PC9 cells were treated with free gefitinib, SUV, SUV-G, SUV-T, and SUV-G+T with or without the BBB for 48 h; (**B**) PC9 were treated with free gefitinib, SUV-Mal, SUV-RF, and SUV-TAT with or without the BBB for 48 h; and (**C**) bEnd.3 cells were treated with free gefitinib, SUV-Mal, SUV-RF, and SUV-TAT for 48 h. Cell viability was determined using the SRB assay. Values are the mean ± SEM. (*n* = 3). * *p* < 0.05 compare to the Gef, ^†^ *p* < 0.05 compare to the SUV, ^‡^ *p* < 0.05 compare to the SUV-Mal, ^§^ *p* <0.05 compare to the SUV-TAT by Student′s *t*-test analysis.

The results showed that the direct cytotoxicity of 15 nM gefitinib on PC9 cells without the BBB (no bEnd.3 cells but with the empty transwell insert) decreased the viability of PC9 cells to 68.81% ± 3.20% (Figure 5A). The cytotoxic effect of gefitinib across the bEnd.3 cells on PC9 cells was dramatically reduced by existence of the BBB and thus the viability of PC9 cells returned to 90.22% ± 1.95% (Figure 5A). Gefitinib in the formulations of SUV, SUV-G, and SUV-T did not further improve the cytotoxicity of gefitinib (Figure 5A). However, gefitinib encapsulated in SUV-G+T across the BBB slightly diminished viability of PC9 cells more than that of free gefitinib or SUV (both *p* < 0.05; Figure 5A).

### 2.6. Cytotoxicity of Gefitinib in SUV-Mal, SUV-TAT, or SUV-RF across the BBB on PC9 Cells

**Figure 6:**
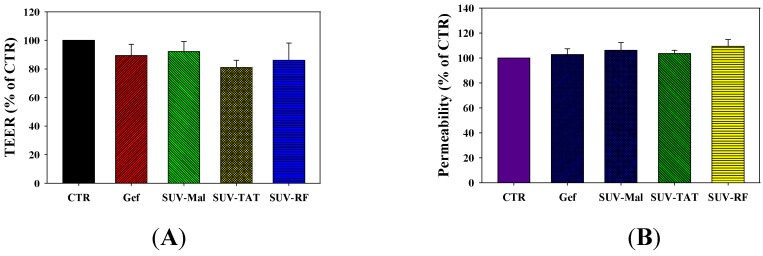
Effect of SUV-RF on the barrier integrity of the BBB model: (**A**) transendothelial electrical resistance after 48-h treatment of free gefitinib, SUV-Mal, SUV-RF, and SUV-TAT on bEnd.3 cells; and (**B**) permeability of sodium fluorescein (SF) across bEnd.3 cells after 48-h treatment of free gefitinib, SUV-Mal, SUV-RF, and SUV-TAT. Values are the mean ± SEM (*n* = 4).

Similarly, gefitinib in the formulations of SUV-Mal and SUV-TAT (one well-known cell penetrating peptide) showed no significant difference from cytotoxicity of free gefitinib (both *p* > 0.05; Figure 5B). Here, maleimide (Mai) is a linker conjugated on the DSPE-PEG for binding to the SH group of TAT and RF. Nevertheless, SUV-RF (one novel cell penetrating peptide) across the BBB significantly further reduced viability of PC9 cells than that of free gefitinib or SUV-Mal (both *p* < 0.05; Figure 5B). Gefitinib in free form or incorporated in SUV-Mal, SUV-TAT, or SUV-RF displayed no cytotoxicity to bEnd.3 cells (Figure 5C) and did not affect the TEER (Figure 6A) of BBB model and permeability of sodium fluorescein across the bEnd.3 monolayer (Figure 6B).

### 2.7. Quantitative Analysis of Cellular Uptake and Transcytosis Mechanisms of SUV-RF

**Figure 7:**
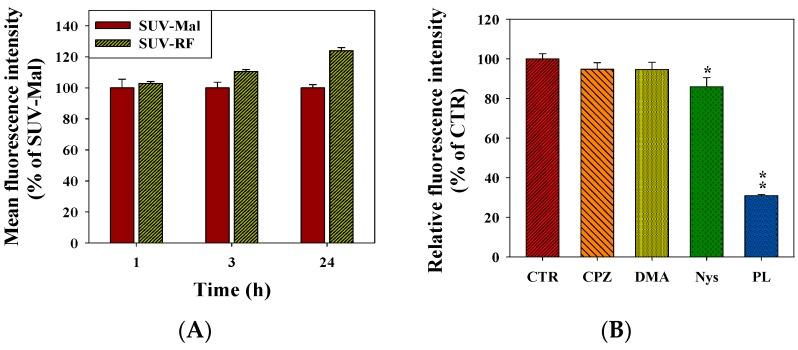

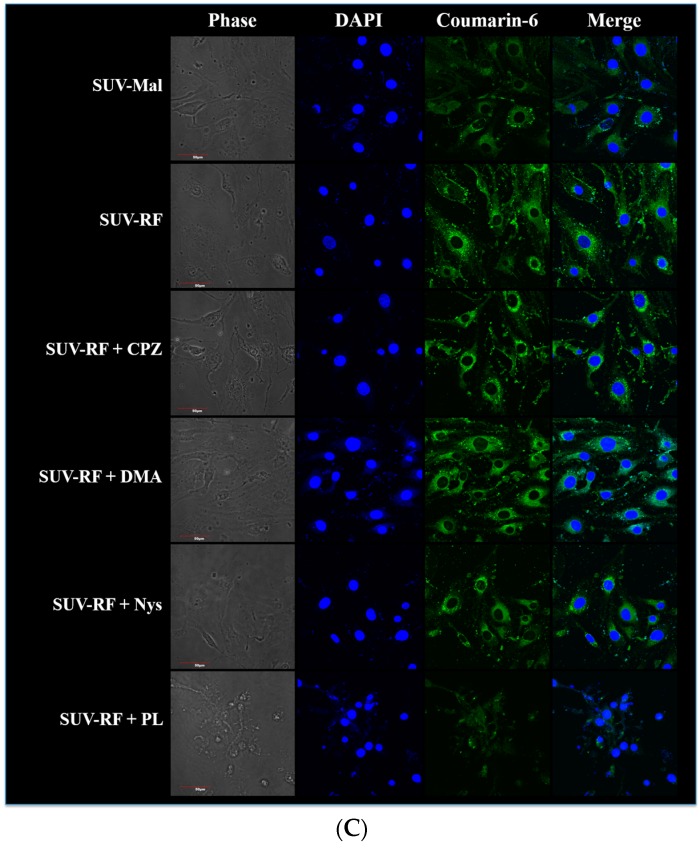
The cellular uptake mechanisms of SUV-RF. bEnd.3 cells were pre-treated with various endocytosis inhibitors including CPZ (10 µM, clathrin-mediated endocytosis inhibitor), DMA (20 µg/mL, macropinocytosis inhibitor), Nys (10 µg/mL, caveolae-mediated endocytosis inhibitor) and PL (250 µg/mL, positive-charged pathway) for 30 min and then incubated with coumarin-6-loaded SUV-RF for 90 min. Two methods including: flow cytometry (**A**,**B**); and confocal laser scanning microscope (**C**) were used to detect the relative fluorescence intensity and uptake images of internalized coumarin-6-loaded SUV-RF after treatment of different endocytosis inhibitors. Values are the mean ± SEM. (*n* = 3). * *p* < 0.05, ** *p* < 0.01 compared to control by Student′s *t*-test analysis. Abbreviations: CPZ, chlopromazine hydrochloride; DMA, 5-(*N*,*N*-Dimethyl) amiloride hydrochloride; Nys, nystatin; PL, poly-lysine. Bar = 50 μm.

Because SUV-RF demonstrated the highest cytotoxicity across the BBB on PC9 cells among all the formulations used in this study, we thus chose this formulation for further investigation. The cellular uptake of coumarin-loaded SUV-Mal and SUV-RF by bEnd.3 cells was monitored by flow cytometer and confocal laser scanning microscope (CLSM), as exhibited in Figure 7. After incubation of cells with SUV-Mal and SUV-RF for 0.5, 3, and 24 h, the mean fluorescence intensity of different treatment groups was normalized relatively to the value of SUV-Mal at 0.5 h. After 3 h uptake, relative fluorescence intensity of SUV-RF was significantly higher than that of SUV-Mal (*p* < 0.05; Figure 7A). As treatment period was increased to 24 h, SUV-RF displayed more intracellular accumulation than that of SUV-Mal (*p* < 0.01; Figure 7A). To investigate the mechanism underlying the intracellular uptake of SUVs, various endocytosis inhibitors were pretreated for 30 min to detect the internalization pattern of SUV-RF. When nystatin, an inhibitor of caveolae-mediated endocytosis was added, the relative uptake percentage was decreased to 85.87% ± 4.63% for SUV-RF, as demonstrated in Figure 7B. This indicated that the internalization of SUV-RF was partially mediated via caveolae-mediated transcytosis. Moreover, the pre-treatment of bEnd.3 cells by poly-lysine for 30 min before the addition of SUV-RF, an inhibitor of adsorptive transcytosis, the relative uptake percentage was further diminished to 30.91% ± 0.65% for SUV-RF (Figure 7B). This revealed that the cellular uptake of SUV-RF was mainly mediated through adsorptive transcytosis via electrostatic interaction between cationic RF and anionic membrane surface of bEnd.3 cells. In contrast, the uptake of SUV-RF had no significant difference before and after addition of 5-(*N*,*N*-Dimethyl) amiloride, an inhibitor of micropinocytosis, and chlorpromazine, an inhibitor of clathrin-mediated endocytosis, suggesting no involvement of micropinocytosis and clathrin-mediated endocytosis in the cellular uptake of SUV-RF across the bEnd.3 cells.

The cellular uptake of coumarin-loaded SUV-Mal and SUV-RF by bEnd.3 cells was also verified by CLSM (Figure 7C). The cell nucleus was stained with 4′,6-diamidino-2-phenylindole (DAPI) (blue) for comparison. After incubation of cells with SUV-Mal or SUV-RF for 90 min, the green fluorescence, including green spots was concentrated and distributed around the cell nucleus. SUV-RF appeared to display intensely additional green fluorescence when compared to SUV-Mal after uptake of these two SUV for 90 min, separately (Figure 7C). The pretreatment of poly-lysine and nystatin inhibited the internalization of SUV-RF onto the bEnd.3 cells, especially signified by the lower accumulation of SUV-RF around the nucleus by the addition of poly-lysine (Figure 7C). This also supported the uptake mechanisms of SUV-RF via adsorptive- and caveolae-mediated transcytosis provided by the flow cytometric study (Figure 7A,B).

## 3. Discussion

The major obstacle to treat BM is the limited penetration of chemotherapy across the blood–brain barrier (BBB). Gefitinib, a first generation EGFR-TKI for treating NSCLC, has low cerebrospinal fluid-to-plasma ratio of about 0.3%–1.3% in patients with BM [3]. This limits the use of gefitinib in treating BM. We thus proposed to establish an in vitro model to mimic the BBB and confirmed the integrity of this BBB monolayer (Figure 3). The nontoxic concentrations of compounds were screened on bEnd.3 cells (Figure 4). We have designed liposomes with two different modifications: SUV-G+T and SUV-RF. The liposomal preparations demonstrated acceptable physicochemical characteristics (Figure 1 and Figure 2, Table 1).

GSH receptors at the BBB interface offer an option in targeting these receptors by GSH-coated nanoparticles to escalate the active or passive transcytosis of therapeutic agents to the brain [12,13]. GSH-PEGylated liposomes have been effectively applied as a brain drug delivery platform for the improvement of numerous treatments. One of the most developed GSH-liposomes is 2B3-101, which is PEGylated liposomal doxorubicin modified with GSH for the treatment of multiple brain cancer indications [15]. We have prepared liposomes with surface coating by GSH in this study. Moreover, liposomes were further modified with Tween 80 to evaluate their BBB penetration ability. Such a design has been found to cross the BBB by augmenting the plasma adsorption of apolipoprotein E or B on the nanoparticle surface for increasing binding to the low density lipoproteins (LDL) receptor [18]. Furthermore, nanoparticles modified with Tween 80 may circumvent P-gp transporters, thus intensifying uptake of nanoparticles across the BBB [19,20]. In this study, our results mildly supported that gefitinib-encapsulated in liposomes modified with GSH and Tween 80 demonstrated greater cytotoxicity than that of free gefitinib or gefitinib-SUV (both *p* < 0.05; Figure 5A). The concentrations of GSH and Tween 80 used in this study were nontoxic to bEnd.3 cells (Figure 4A,B).

**Figure 8:**
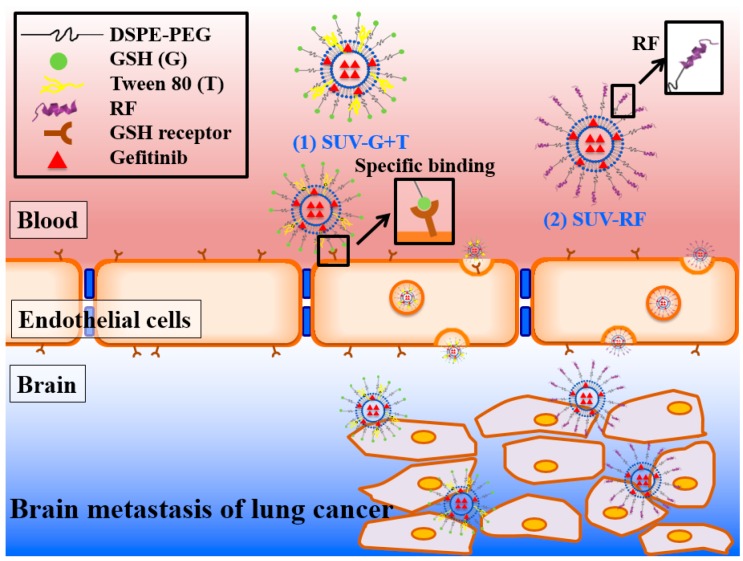
Schematic illustration of delivery of gefitinib-loaded PEGylated liposomes across the BBB. SUV-G+T and SUV-RF may bind to receptors expressed on the surface of brain endothelial cells, enhance gefitinib transport across the BBB, and target to tumor sites.

Another approach is to prepare liposomes with surface conjugation to RF for evaluating their BBB penetration effect. RF, a cell-penetrating peptide, possesses 17 amino acid residues and exhibits the structure of α-helix. This CPP bears six positive charges and has amphipathic property [27]. It has been reported that cationic α-helical CPP, including RF may interact with negatively charged extracellular glycosaminoglycans (GAGs) such as heparan sulfate (HS) via direct translocation and micropinocytosis [33]. The direct translocation is an endocytosis-independent process that the CPPs transport across the cell membrane via the barrel-stave model, inverted micelle model, or carpet model [34,35]. RF bearing Arg and Phe displayed a greater capacity in binding to HS and causing HS-clustering, accounting for a superior cellular uptake ability via electrostatic and hydrophobic interactions when compared with peptides possessing Glu or Ala in HeLa and A549 cells [33]. RF also showed selective cytotoxicity to cancer cells, but exhibited lower cellular uptake into normal cells including mouse fibroblast 3T3-L1 in comparison to TAT [25,33]. Our flow cytometric and CLSM results demonstrated that the endocytosis pathways of the uptake of SUV-RF into bEnd.3 cells was mainly through adsorptive transcytosis-mediated mechanism via electrostatic interaction of positively-charged Arg and Lys in RF with negatively charged glycoproteins of cell membrane and partially through caveolae-mediated transcytosis. Such PEGylated liposomes linked with RF showed efficacy to cross the BBB model to reduce the viability of PC-9 cells and displayed the advantage of low toxicity to bEnd.3 cells. The proposed schematic illustration of delivery of gefitinib-loaded PEGylated liposomes across the BBB is shown in Figure 8.

## 4. Materials and Methods

### 4.1. Materials

Gefitinib was a kind gift or obtained from AstraZeneca, Alderley Park, UK or (AstraZeneca, Macclesfield, Cheshire, UK). RF with the amino acid sequence of GLKKLARLFHKLLKLGC was purchased from Kelowna Biotech (Taipei, Taiwan) at >95% purity. Cholesterol was purchased from Sigma-Aldrich (St. Louis, MO, USA). DSPC and DSPE-PEG2000-maleimide were obtained from Avanti Polar Lipids, Inc. (Alabaster, AL, USA). All cell culture medium and reagents were bought from Promega (Madison, WI, USA), Invitrogen (Carlsbad, CA, USA), Gibco BRL (Grand Island, NY, USA), or Hyclone (Logan, UT, USA). All other chemical reagents were obtained from either Merck (Darmstadt, Germany) or Sigma-Aldrich.

### 4.2. Preparation of PEGylated Liposomal Gefitinib-Formulations

DSPC, cholesterol, and DSPE-PEG-NH2 or DSPE-PEG-maleimide (for preparation of SUV-RF) were dissolved in chloroform and methanol (2:1). The resulting lipid solution was then dried using an evaporator. The lipid film was resuspended in 250 mM ammonium sulfate for 20 min under ultrasonication. The dispersion was extruded through a 0.1 µm membrane for four times. The product was ultrasonicated for 10 min. Gefitinib was loaded using dialysis against 500 mM sucrose and vortex for 1 h. The final gefitinib-containing liposomes were then dialyzed against pH 7.4 PBS.

The resultant liposomes were further reacted with RF at molar ratio of DSPE-PEG-maleimide: peptide (TAT or RF) = 1:1.5 for 24 h at room temperature. To obtain GSH or Tween 80 modification, liposomal solution was mixed with 0.84% GSH (*w*/*v* %) or 400 µM Tween 80. The mixture was ultrasonicated at room temperature for 30 min to allow a maximal GSH or Tween 80 modification. After GSH coating, the final liposomes were centrifuged at 15,000 rpm for 5 min (4 °C) through an Amicon Ultra-4 Centrifuge Filter (10,000 MWCO, Millipore Corp., Bedford, MA, USA). The supernatant was collected and the pH value was adjusted to 7.4. The above supernatant or GSH standard were then mixed with 6 mM Ellman’s reagent (5,5′-dithiobis-2-nitrobenzoic acid (DTNB)). The absorbance of the formed yellow 5-thio-2-nitrobenzoic acid (TNB) proportional to total GSH concentration was measured at 412 nm at 25 °C using a Tecan Infinite 200^®^ PRO multimode microplate reader (Männedorf, Switzerland). According to our calculation, the GSH coating percentage was 59.70%. Thus, the final concentration of GSH was determined to be 0.5% (*w*/*v* %). Descriptions of these liposomal formulations are demonstrated in Table 1.

### 4.3. Characterization of PEGylated Liposomal Gefitinib-Formulations: Encapsulation Efficiency, Size Distribution, Zeta Potential, and Transmission Electron Microscopic Image

Unbound gefitinib was separated from the loaded liposomes by filtration and centrifugation. Gefitinib in the filtrate was analyzed by HPLC. A L7100 chromatography pump (Hitachi, Tokyo, Japan) equipped with an automated injector (Primaide 1210), a 5-μm Luna C18 liquid chromatography (LC) column (Phenomenex, Torrance, CA, USA) and an ultraviolet L2400 detector (Hitachi, Tokyo, Japan) was used for gefitinib analysis. The mobile phase was composed of 0.5% KH_2_PO_4_, acetonitrile, and methanol (55:25:20, *v*/*v*/*v*). The mixture was pre-degassed using a sonicator. The flow rate was 1 mL/min at room temperature. The peak area of gefitinib was calculated and compared with the calibration curve for quantitation. Each experiment was performed in quadruplicate.

EE% was calculated by the Equation (1) as shown below.

(1) $$EE\%=\left(\frac{W_{e}-W_{f}}{W_{e}}\right)\times 100\%$$

where *W*_e_ is the weight of added gefitinib and *W*_f_ is the weight of gefitinib in the filtrate.

The size distribution and ζ potential of liposomes were measured using a Zetasizer Nano ZS90 (Malvern Instruments Ltd., Malvern, Worcestershire, UK) at 25 °C with a scattering angle of 90.0 °. Data was calculated by a cumulant method with Zetasizer family software v7.11 to obtain polydispersity index. Records were analyzed from four individual measurements.

The shape and particle morphology of SUV was observed under TEM. One drop of the sample solution was mounted onto a carbon-coated copper grid (300 mesh) for one minute at room temperature. The grid was air-dried at room temperature for five minutes. One drop of 1% phosphotungstic acid was then dripped onto the grid for five minutes. The preparation was air-dried for 30 min and then observed using a JEM 2000EX II transmission electron microscope (JEOL, Ltd., Tokyo, Japan) at 100 kV.

### 4.4. In Vitro Release of Gefitinib from SUV

Gefitinib release from SUV was measured using a dynamic release assay under sink conditions. An aliquot of each gefitinib formulation (1 mL) was put inside a dialysis bag and firmly wrapped. Then the dialysis bags were immersed in 500 mL of PBS in an incubator at 37 °C for 24 h with mild stirring at a rate of 100 rpm for maintaining a uniform drug concentration in the medium. At 1, 2, 4, 8, 12, and 24 h, the samples of 0.1 mL were removed and stored at −20 °C until analysis. With each sampling, the medium was replaced with pre-warmed PBS to maintain the total volume constant. The release of gefitinib from these preparations was sampled up to 24 h. The samples were centrifuged and the concentration of gefitinib in the collected supernatants was detected by HPLC. The cumulative gefitinib released percent was then determined.

### 4.5. Culture of Human Lung Adenocarcinoma PC-9 Cells and Murine Brain Endothelial bEnd.3 Cells

PC-9 cells are a human lung adenocarcinoma cell line harboring a deletion in exon 19 of EGFR [6]. This cell line was provided by Chih-Hsin Yang of National Taiwan University. PC-9 cells were maintained in Roswell Park Memorial Institute-1640 Medium (RPMI-1640; Hyclone) containing 10% fetal bovine serum (FBS), 1% l-glutamine (l-G), 1% penicillin-streptomycin-amphotericin B (PSA) at pH 7.2–7.4. bEnd.3 cells (murine brain endothelial cells) are provided by Prof. Chuen-Mao Yang of Chang Gung University. This cell line was incubated in Dulbecco′s Modified Eagle Medium with nutrient mixture F-12 (D-MEM/F12; Gibco) composed of 10% FBS, 1% l-glutamine, 1% PSA, 2.4 g/L sodium bicarbonate, and 20 mM HEPES. All the cell culture reagents were purchased from Gibco or HyClone. The culture was maintained at 37 °C under a humidified atmosphere of 5% CO_2_ and 95% air.

### 4.6. Establishment of In Vitro BBB Model: Morphology of Barrier Integrity, Transendothelial Electrical Resistance (TEER) Measurement, and Permeability Measurement

bEnd.3 cells (5.5 × 10^4^ cell/insert) were seed on 24-well tissue culture inserts (0.4 µm, Transwell, polyester membrane). After culture of different days, the monolayers were evaluated for the following experiments to check the integrity of the BBB model. Morphology of the BBB model was observed by staining bEnd.3 cells with crystal violet and cell image was captured under a Nikon Diaphot Inverted Tissue Culture Microscope (Diaphot 300, Nikon, Tokyo, Japan).

TEER was measured by a Millicell ERS-2 volt-ohm meter (Millipore Corp.) and a STX01 chopstick-style electrode (Millipore). To calculate TEER (Ω·cm^2^), electrical resistance across a collagen IV-coated insert without cells (*R*_blank_) was subtracted from the readings obtained on inserts with cells (*R*_total_) and this value was multiplied by the surface area (A) of the insert (0.336 cm^2^). The TEER of the cell monolayers was calculated according to the equation:

(2) $$TEER=(R_{\mathrm{total}}-R_{\mathrm{blank}})\times A\;(\Omega \cdot {\mathrm{cm}}^{2})$$

where *R*_total_ is the resistance measured, *R*_blank_ is resistance of control filters without cells and A is the surface area of filter (0.336 cm^2^).

After incubation, the upper wells with 100 µL of fresh serum-free medium containing 10 µg/mL of sodium fluorescein (376 Da; paracellular marker) or 0.09 mg/mL of fluorescein isothiocyanate-conjugated dextran (70 KDa FITC-dextran; paracellular marker) were inserted into new lower wells containing 0.6 mL of fresh serum-free medium. After incubation in the dark at 37 °C for 1 h, 50 µL of the lower well media was sampled, and fluorescence intensity of sodium fluorescein or FITC-dextran was measured with excitation at 460 and 494 nm and emission at 515 and 521 nm, respectively, using a Tecan microplate reader. The relative permeability changes were calculated corresponding to coated inserts without cells, which represented a reference for maximal permeability [36].

(3) $$Permeability\;(\%\;\mathrm{of}\;\max)=\frac{{\mathrm{fluorescence}\;\mathrm{intensity}}_{\mathrm{coated}\;\mathrm{inserts}\;\mathrm{with}\;\mathrm{cells}}-{\mathrm{fluorescence}\;\mathrm{intensity}}_{\mathrm{medium}}}{{\mathrm{fluorescence}\;\mathrm{intensity}}_{\mathrm{inserts}\;\mathrm{without}\;\mathrm{cells}}-{\mathrm{fluorescence}\;\mathrm{intensity}}_{\mathrm{medium}}}$$

### 4.7. Cell Viability by the SRB Assay

After overnight seeding of PC-9 and bEnd.3 cells in 96-well flat-bottomed plates at density of 4 × 10^3^ cells/well and 8 × 10^3^ cells/well, different concentrations of gefitinib were added in the culture medium for the indicated time. The cytotoxic effects were determined by sulforhodamine B assay [37]. Cell viability was determined by dividing the absorbance values of treated cells to that of cells of medium control.

### 4.8. Endocytic Uptake Mechanisms of SUV-RF

bEnd.3 cells were pre-treated with various endocytosis inhibitors, such as chlopromazine hydrochloride (CPZ; 10 µM, clathrin-mediated endocytosis inhibitor), 5-(*N*,*N*-dimethyl) amiloride hydrochloride (DMA; 20 µg/mL, micropinocytosis inhibitor), nystatin (Nys; 10 µg/mL, caveolae-mediated endocytosis inhibitor) and poly-lysine (PL; 250 µg/mL, inhibitor of adsorptive transcytosis) for 30 min and then incubated with coumarin-6-loaded SUV-RF for 90 min [38]. Two methods including flow cytometry and confocal laser scanning microscope (CLSM) were used to detect the relative fluorescence intensity and uptake images of internalized coumarin-6-loaded SUV-RF after treatment of different transcytosis inhibitors. For flow cytometric analysis, after incubation of cells with SUV-Mal and SUV-RF for 0.5, 3, and 24 h, the cells were detached by Accumax, collected, and suspended in PBS at 37 °C. Flow cytometric analysis was then carried out using a FACSCalibur flow cytometer (BD Biosciences, San Jose, CA, USA) equipped with an argon ion laser and operated at 488 nm. Fluorescence was measured through a 520 nm FL1 filter (515–545 nm) for coumarin-6 and fluorescence signals were collected on a logarithmic scale. Data acquisition and analysis were performed using commercial BD FACStation™ software (BD Biosciences). At least 10,000 cells were analyzed in each sample. Within each experiment, determinations were performed in triplicate.

### 4.9. Intracellular Uptake of SUV-RF by Confocal Laser Scanning Microscope (CLSM)

After treatment with various transcytosis inhibitors for 30 min, the cells were further incubated with coumarin-6 (in green)-loaded SUV-RF at 37 °C for 90 min. The cells were then rinsed using PBS. The images were taken using a confocal laser-scanning microscope (Olympus FV10i, Olympus America Inc., Center Valley, PA, USA) with excitation at 490 nm and emission at 520 nm. The cells were also stained with DAPI (in blue) in the nucleus for comparison. At least 3 photos were taken in each sample. The representative image of each treatment is exhibited.

### 4.10. Statistical Analysis

Experimental data were analyzed by Student′s *t*-test and expressed as the mean ± SEM. Statistical significance was set at *p* < 0.05.

## 5. Conclusions

Taken together, cell selective CPP-conjugated SUV-RF shed light on improving gefitinib delivery across the BBB via modulating the transcytosis pathway(s). We expect that gefitinib loaded SUV-RF will potentially become a more promising brain delivery platform due to their ability to increase penetration across the BBB and enhance targeting to the tumor cells. This liposomal formulation may reduce the off-target side effects to normal brain cells. The special and serious situation of brain metastases highlights the necessity for a multifunctional approach integrating an effective delivery system to carry gefitinib as a potential nanomedicine to enhance the clinical efficacy of target therapy.

## Figures and Tables

**Table 1:** Characterization of gefitinib-loaded liposomes modified with glutathione (GSH), Tween 80, or RF **^a^**.

Formulation	Description	Particle Size (nm)	PDI ^b^	ζ Potential (mV)
SUV-Mal	Small unilamellar vesicles of DSPC, cholesterol, and DSPE-PEG2000-maleimide	95.5 ± 2.2	0.16 ± 0.01	−3.38 ± 0.78
SUV-RF	Small unilamellar vesicles of DSPC, cholesterol, DSPE-PEG2000-maleimide, and conjugated with RF	147.1 ± 3.9	0.10 ± 0.02	−3.42 ± 0.64
SUV	Small unilamellar vesicles of DSPC, cholesterol, and DSPE-PEG-NH2	105.5 ± 6.6	0.12 ± 0.03	−3.25 ± 0.27
SUV-G	Small unilamellar vesicles of DSPC, cholesterol, DSPE-PEG-NH2, and coated with GSH	102.3 ± 6.3	0.06 ± 0.01	−1.70 ± 0.16
SUV-T	Small unilamellar vesicles of DSPC, cholesterol, DSPE-PEG-NH2, and modified with Tween 80	85.8 ± 3.7	0.13 ± 0.03	−3.09 ± 0.75
SUV-G+T	Small unilamellar vesicles of DSPC, cholesterol, DSPE-PEG-NH2, and modified with GSH and Tween 80	94.2 ± 0.7	0.26 ± 0.01	−3.82 ± 0.85

**^a^** RF, one cell-penetrating peptide; **^b^** PDI, polydispersity index.
